# Transversus Abdominis Plane Block With Dexamethasone and Dexmedetomidine Decreases Opioid Consumption, Length of Stay, and Ileus After Laparoscopic Colorectal Resection

**DOI:** 10.7759/cureus.106684

**Published:** 2026-04-08

**Authors:** Shafic Abdulkarim, Ammar Saed Aldien, Julie Savard, Nancy Morin, Carol-Ann Vasilevsky, Allison J Pang

**Affiliations:** 1 Department of Surgery, McGill University, Jewish General Hospital, Montreal, CAN; 2 Faculty of Medicine and Health Sciences, McGill University, Montreal, CAN; 3 Division of Colon and Rectal Surgery, Jewish General Hospital, Montreal, CAN

**Keywords:** colorectal surgery, enhanced recovery after surgery (eras), opioid consumption, postoperative pain control, transversus abdominis plane (tap) block

## Abstract

Background: This study investigated the impact of transversus abdominis plane (TAP) blocks containing bupivacaine hydrochloride, dexamethasone, and dexmedetomidine on postoperative outcomes following laparoscopic colorectal resections.

Patients and methods: This retrospective cohort study included patients who had undergone elective laparoscopic colorectal resections between 2019 and 2022. Patients received either local wound infiltration or TAP blocks. TAP blocks were comprised of bupivacaine hydrochloride, dexamethasone, and dexmedetomidine. Local infiltration was with bupivacaine alone. Postoperative morphine milligram equivalents (MME) and visual analog scale (VAS) scores were compared. Predictors of MME and length of stay (LOS) were investigated by multiple linear regression.

Results: A total of 195 patients met the inclusion criteria. Among them, 90 (46.1%) received local wound infiltration, while 105 (53.9%) received TAP blocks. Patients in the TAP block group experienced lower postoperative MME at 24 hours (49.9 mg vs. 77.33 mg, p = 0.028) and a shorter LOS of 4.4 ± 2.2 days compared to 6.1 ± 5.1 days (p = 0.001). VAS scores showed statistically similar results between treatment groups at all three postoperative time points. Additionally, TAP blocks were associated with a reduced incidence of postoperative ileus (0% vs. 5%, p = 0.01). Multiple linear regression indicated that TAP blocks were independently associated with lower MME at 24 hours after surgery (β = -31.3, 95% CI: -57.5 to -5.1, p = 0.019) and a shorter LOS (β = -1.9, 95% CI: -3.4 to -0.4, p = 0.013).

Conclusion: TAP blocks using bupivacaine, dexamethasone, and dexmedetomidine are associated with decreased opioid consumption at 24 hours, shorter LOS, and reduced ileus compared to local infiltration alone.

## Introduction

Enhanced Recovery After Surgery (ERAS) protocols are quintessential for improving outcomes in colorectal surgery [1,2] and are associated with faster bowel function recovery, decreased complication and readmission rates, and shorter length of stay (LOS) [3,4]. A key element of these protocols is the use of multimodal analgesia to reduce postoperative opioid use. The transversus abdominis plane (TAP) block was first described by Rafi in 2001 [5] and involved the injection of local anesthetic into the plane between the transversus abdominis and internal oblique muscles. This block has been shown to decrease both postoperative opioid consumption and LOS [6]. For this reason, TAP blocks have been increasingly implemented in postoperative analgesia pathways after various minimally invasive surgeries, including bariatric, colorectal, biliary, and gynecologic surgeries [6,7].

One of the main downsides of using only local anesthetics in TAP blocks is the short duration of action, with the longest duration being approximately four to eight hours when an agent such as bupivacaine is used. To mitigate this short duration of analgesia, dexamethasone and dexmedetomidine have been studied as adjuncts to local anesthetics in these blocks. Both have proven effective in prolonging the duration of action of TAP blocks and enhancing postoperative pain control, with available evidence suggesting that their combination may further extend analgesia by approximately 16-20 hours compared to either agent alone [8,9]. Less is known about the synergistic effects of dexamethasone and dexmedetomidine in TAP blocks. However, this combination is frequently used in orthopedic surgeries and is well-documented in selective nerve blocks [10,11]. Chapman et al. [12] investigated the effects of combining dexamethasone and dexmedetomidine as dual adjuncts in TAP blocks after minimally invasive colorectal surgeries. Their results showed that patients who underwent these blocks had significantly lower postoperative opioid use up to 48 hours after surgery compared to those who received TAP blocks using only local anesthetic and dexamethasone. Although their sample size was small (55 patients), their results were promising.

This study aimed to evaluate whether TAP blocks containing dexamethasone and dexmedetomidine reduce postoperative opioid consumption, LOS, and postoperative ileus compared to local wound infiltration in patients undergoing elective laparoscopic colorectal surgery. We hypothesized that TAP blocks would be associated with reduced opioid use and improved postoperative recovery outcomes.

This article was previously presented as a meeting abstract at the 2023 SAGES (Society of American Gastrointestinal and Endoscopic Surgeons) Annual Meeting, held from March 29 to April 1, 2023, at the Montreal Convention Centre in Montreal, Quebec, Canada.

## Materials and methods

Study population

This retrospective cohort study was conducted at a single university-affiliated tertiary care center with Institutional Review Board (IRB) approval. Patients who underwent elective laparoscopic colorectal resection between 2019 and 2022 for malignant or benign diseases were included. The procedures comprised small bowel resection, ileocolic resection, right hemicolectomy, left hemicolectomy, low anterior resection, subtotal colectomy, and total colectomy. Low anterior resection, sigmoid resection, left hemicolectomy, and proctocolectomy were classified as “Left Colonic Resections,” while ileocolic resection and right hemicolectomy were categorized as “Right Colonic Resections.” Patients were excluded if they had a minimally invasive procedure that converted to open surgery, had been taking preoperative opioids, or had missing data on opioid consumption. Electronic medical records were reviewed for all included patients. The extracted data encompassed age, sex, BMI, American Society of Anesthesiologists (ASA) score, type of resection, stoma creation, specimen extraction site, use of patient-controlled analgesia (PCA), morphine milligram equivalents (MME), visual analog scale (VAS) scores, anastomotic leak, surgical site infection (SSI), ileus requiring nasogastric tube insertion, and LOS.

Patients were grouped based on whether they received local wound infiltration or TAP blocks. Local wound infiltration served as the comparator, as it was the standard method of postoperative analgesia at our institution prior to the adoption of TAP blocks. Following this transition in practice, TAP blocks were increasingly utilized at the discretion of the operating surgeon. Each patient received either local wound infiltration or a TAP block, but not both, with no overlap between groups. Both interventions were typically administered at the completion of the procedure.

Local wound infiltration consisted of bupivacaine at a concentration of 0.25% or 0.5%, with or without epinephrine, with the use of epinephrine at the discretion of the operating surgeon.

TAP blocks were performed by the operating surgeon using a laparoscopic approach and included 0.25% bupivacaine hydrochloride, 5 mg dexamethasone, and 50 mcg dexmedetomidine. The surgeon used the laparoscope to visualize the needle as it was inserted through the abdominal wall, lateral to the rectus abdominis muscle. In our practice, TAP blocks were performed using a long 22-gauge needle (10-15 cm) to allow adequate reach and controlled infiltration of the TAP.

A local anesthetic solution combined with adjunct medications was prepared as a single mixture and administered under direct visualization, with total dosing determined based on the maximum weight-based allowable dose for each patient. The total volume of the TAP block solution was divided equally and administered bilaterally, with half injected on each side of the rectus abdominis muscle. On each side, the injection was typically performed at three points within the TAP plane (superior, mid, and inferior portions) to facilitate distribution of the anesthetic. By targeting the space between the internal oblique and transversus abdominis muscles, the needle was placed within the TAP under direct visualization.

Study outcomes

This study aimed to evaluate whether TAP blocks with dexamethasone and dexmedetomidine reduce postoperative opioid consumption, LOS, and postoperative ileus compared to local wound infiltration in patients undergoing elective laparoscopic colorectal surgery. We hypothesized that TAP blocks would be associated with reduced opioid use and improved postoperative recovery outcomes compared to local wound infiltration.

The primary outcome was postoperative opioid consumption, measured using MME. MME was used to standardize opioid exposure across different agents and routes of administration by converting all opioids into an equivalent morphine dose using established equianalgesic conversion factors.

Postoperative pain was assessed using a 0-10 VAS, a widely validated and commonly used measure of pain intensity [13,14]. Measurements at 0 hours for both MME and VAS were defined as the immediate postoperative period in the post-anesthesia care unit (PACU).

Opioid doses were converted to MME using established conversion factors (e.g., hydromorphone ×4, oxycodone ×1.5), based on commonly referenced guidelines such as those from the Centers for Disease Control and Prevention [15]. This approach ensures consistent aggregation and comparison of total opioid consumption across patients receiving different analgesic regimens [16].

Both VAS scores and MME were measured and compared between study groups at 0, 24, 48, and 72 hours postoperatively.

Secondary outcomes included LOS, 30-day readmission, postoperative ileus (defined as the need for nasogastric tube insertion), SSI, and anastomotic leak.

Statistical analysis

Demographic and clinical data were analyzed as an entire cohort. Continuous variables were expressed as means with standard deviations (SD), and categorical variables were reported as numbers and percentages (%). Univariate analysis of differences between surgical groups was performed by a two-tailed Student’s t-test for continuous variables and a Pearson’s chi-square test for categorical variables. A multiple linear regression determined factors associated with MME and LOS. Covariates for the regression models included age, BMI, procedure performed, hand-assisted surgical approach, ASA (score 3-4), female sex, presence of stoma, and midline incision site. Statistical significance was set at a p-value <0.05. All statistical analyses were performed using RStudio version 1.1.463 (Posit, Boston, MA).

## Results

Demographic, clinical, and operative characteristics

Between 2019 and 2022, 338 laparoscopic colorectal resections were performed for benign and malignant disease. Of these, 195 patients met the inclusion criteria. The demographic and clinical characteristics are shown in Table 1. A total of 90 (46.1%) patients received local wound infiltration, and 105 (53.9%) received TAP blocks. The two intervention groups had a similar distribution of age and BMI. Most patients in both groups had an ASA score of 1 or 2 (61 (67.8%) vs. 78 (74.3%), p = 0.312). Compared to the local wound infiltration group, patients in the TAP block group were more likely to be male (42 (46.7%) vs. 64 (61.0%), p = 0.046).

**Table 1:** Demographic and clinical characteristics of the study population. TAP = transversus abdominis plane; SD = standard deviation; BMI = body mass index; ASA = American Society of Anesthesiologists. Values are presented as mean ± standard deviation or number (%), as appropriate. Continuous variables were compared using Student’s t-test, and categorical variables were compared using the chi-square test or Fisher’s exact test, as appropriate.

Variable	Local wound infiltration (n = 90), Mean ± SD or N (%)	TAP block (n = 105), Mean ± SD or N (%)	P-value
Age (years)	64.5 (± 15.5)	63.6 (± 14.2)	0.671
Sex			0.046
Female	48 (53.3%)	41 (39.0%)	
Male	42 (46.7%)	64 (61.0%)	
BMI (kg/m²)	26.6 (± 5.3)	27.1 (± 7.8)	0.561
ASA			0.312
1-2	61 (67.8%)	78 (74.3%)	
3-4	29 (32.2%)	27 (25.7%)	
Left colonic resection	53 (58.8%)	61 (58.1%)	0.690
Surgical approach			0.800
Completely laparoscopic	85 (94.4%)	100 (95.2%)	
Hand-assisted laparoscopy	5 (5.6%)	5 (4.8%)	
Extraction site: Pfannenstiel	64 (71.1%)	76 (72.4%)	0.960
Stoma formation	16 (17.8%)	17 (16.2%)	0.770

More than half of the patients in each group underwent left colonic resections, with no significant difference between the groups (53 (58.8%) vs. 61 (58.1%), p = 0.69). The majority of procedures in both groups were performed completely laparoscopically compared to hand-assisted laparoscopy (85 (94.4%) vs. 100 (95.2%) and 5 (5.6%) vs. 5 (4.8%), respectively; p = 0.80). The most common extraction site was the Pfannenstiel incision, which was similar between both groups (64 (71.1%) vs. 76 (72.4%), p = 0.96). Stoma formation rates were also comparable (16 (17.8%) vs. 17 (16.2%), p = 0.77).

Postoperative outcomes

The primary outcome of this study was the postoperative MME at 24 hours. Univariate analysis showed that the MME at 24 hours was significantly lower in patients receiving TAP blocks compared to those receiving local wound infiltration (49.92 mg vs. 77.35 mg, p = 0.029). However, statistical significance was not achieved for differences in MME between the groups at 48 hours and 72 hours postoperatively (31.49 mg vs. 51.53 mg, p = 0.123, and 12.76 mg vs. 27.14 mg, p = 0.191, respectively) (Table 2). The VAS was also utilized to complement the postoperative MME. Both intervention groups recorded similar pain scores at 0 hours (4.68 vs. 6.91, p = 0.128), 24 hours (4.67 vs. 6.97, p = 0.259), 48 hours (4.03 vs. 4.47, p = 0.676), and 72 hours (3.01 vs. 2.66, p = 0.422), despite the overall lower use of MME in the TAP block group (Table 2). Notably, 102 (52.3%) of the study cohort utilized a PCA postoperatively, with a similar proportion of patients receiving PCA between groups (43 (47.8%) vs. 59 (56.2%), p = 0.15). A subgroup analysis excluding patients who received PCA demonstrated that patients in the TAP block group still had lower MME at 24 hours (21.25 mg vs. 29.3 mg, p = 0.20) and similar VAS scores at 24 hours (4.76 vs. 4.53, p = 0.68), 48 hours (4.04 vs. 3.22, p = 0.17), and 72 hours (3.22 vs. 2.97, p = 0.71) (data not shown).

**Table 2:** Pain control-related outcomes of local wound infiltration versus transversus abdominis plane (TAP) block. Values are presented as mean ± standard deviation. Continuous variables were compared between groups using Student’s t-test. MME = morphine milligram equivalents; VAS = visual analog scale (range from 0 to 10). MME 0 hour = morphine milligram equivalents administered in the immediate postoperative period. VAS 0 hour = visual analog scale score recorded in the immediate postoperative period.

Outcome (Time point)	Local wound infiltration (n = 90), Mean ± SD	TAP block (n = 105), Mean ± SD	P-value
MME 0 hour	48.32 (± 56.84)	32.67 (± 29.70)	0.015
MME 24 hours	77.35 (± 109.27)	49.92 (± 60.75)	0.029
MME 48 hours	51.53 (± 124.42)	31.49 (± 42.89)	0.123
MME 72 hours	27.14 (± 105.14)	12.76 (± 25.12)	0.191
VAS 0 hour	6.91 (± 2.72)	4.68 (± 2.88)	0.128
VAS 24 hours	6.97 (± 2.65)	4.67 (± 2.40)	0.259
VAS 48 hours	4.47 (± 2.10)	4.03 (± 2.37)	0.676
VAS 72 hours	2.66 (± 2.05)	3.01 (± 2.99)	0.422

Other postoperative and secondary outcomes are outlined in Table 3. There were no significant differences between the groups in 30-day readmission (3/101 (3.0%) vs. 0/94 (0%), p = 0.099), SSI (2/101 (2.0%) vs. 0/94 (0%), p = 0.180), and anastomotic leak (3/101 (3.0%) vs. 0/94 (0%), p = 0.099). The TAP block group had a shorter LOS (4.4 ± 2.2 days vs. 6.1 ± 5.1 days, p = 0.001). Additionally, postoperative ileus was lower in the TAP block group (0/105 (0%) vs. 5/90 (5.6%), p = 0.016); however, the overall ileus rate in the entire study cohort remained low.

**Table 3:** Postoperative outcomes between the two study groups. * The total number of patients in the TAP block group is 101 for this table, rather than the 105 enrolled, because complete outcome data were unavailable for four patients. Percentages were calculated based on the patients with available data. Analyses were performed using complete-case methodology, excluding patients with missing outcome data. TAP = transversus abdominis plane; SD = standard deviation; LOS = length of stay; SSI = surgical site infection; NGT = nasogastric tube. Values are presented as mean ± standard deviation or number (%), as appropriate. Continuous variables were compared using Student’s t-test, and categorical variables were compared using the chi-square test or Fisher’s exact test, as appropriate.

Outcome	Local wound infiltration (n = 90), Mean ± SD or N (%)	TAP block (n = 101)*, Mean ± SD or N (%)	P-value
LOS	6.19 (± 5.1)	4.4 (± 2.22)	0.001
30-day readmission			0.099
No	90 (100%)	98 (97.03%)	
Yes	(0%)	3 (2.97%)	
SSI			0.180
No	90 (100%)	99 (98.02%)	
Yes	(0%)	2 (1.98%)	
Anastomotic leak			0.099
No	90 (100%)	98 (97.03%)	
Yes	(0%)	3 (2.97%)	
Ileus requiring NGT			0.016
No	85 (94.44%)	101 (100%)	
Yes	5 (5.56%)	(0%)	

Factors associated with primary and secondary outcomes

After adjusting for demographic, clinical, and operative factors, TAP blocks were independently associated with lower MME at 24 hours post surgery (β = -31.3, 95% CI: -57.5 to -5.1, p = 0.019) (Table 4), as well as a shorter LOS (β = -1.9, 95% CI: -3.4 to -0.4, p = 0.013) (Table 5). Similarly, in a multivariate analysis of the subgroup of patients who did not receive PCA, TAP blocks remained independently associated with lower MME at 24 hours (β = -18.7, 95% CI: -34.2 to -3.2, p = 0.019) and shorter LOS (β = -1.3, 95% CI: -2.5 to -0.2, p = 0.021) (data not shown). A binomial logistic regression to determine factors associated with ileus was not feasible due to the low number of ileus cases in our patient cohort.

**Table 4:** Multiple linear regression of demographic, clinical, and operative variables according to morphine milligram equivalents at 24 hours. TAP = transversus abdominis plane; BMI = body mass index; ASA = American Society of Anesthesiologists. Estimate (B) coefficients represent the adjusted change in postoperative opioid consumption measured in morphine milligram equivalents (MME) at 24 hours. Continuous parameters are modeled per one-unit increase (age per year; BMI per kg/m²). Categorical parameters are presented relative to their reference categories: local wound infiltration, non-left colonic resection, completely laparoscopic approach, male sex, ASA grade 1-2, no stoma, and Pfannenstiel extraction site. P-values were calculated using Wald tests from multivariable linear regression models.

Parameter	Estimate (β)	95% confidence limits		P-value
TAP block	-31.3	-57.5	-5.1	0.019
Age	-1.8	-2.5	-1.1	<0.001
BMI	0.6	-1.8	3.1	0.628
Left colonic resection	8.6	-64.9	82.2	0.818
Hand-assisted	-2.9	-34.5	28.7	0.856
ASA (3-4)	24.6	-11.3	60.6	0.180
Sex (F)	-28.7	-56.8	-0.6	0.046
Stoma (Yes)	24.2	-19.2	67.6	0.274
Midline incision	-26.1	-47.2	5.0	0.015

**Table 5:** Multiple linear regression of demographic, clinical, and operative variables according to the length of hospital stay (LOS). TAP = transversus abdominis plane; BMI = body mass index; ASA = American Society of Anesthesiologists. Estimate (β) coefficients represent the adjusted change in hospital length of stay measured in days. Continuous parameters are modeled per one-unit increase (age per year; BMI per kg/m²). Categorical parameters are presented relative to their reference categories: local wound infiltration, non-left colonic resection, completely laparoscopic approach, male sex, ASA grade 1-2, no stoma, and Pfannenstiel extraction site. P-values were calculated using Wald tests from multivariable linear regression models.

Parameter	Estimate (β)	95% confidence limits		P-value
TAP block	-1.9	-3.4	-0.4	0.013
Age	0.04	0.02	0.10	0.18
BMI	0.07	-0.12	0.26	0.47
Left colonic resection	1.6	-5.2	8.5	0.64
Hand-assisted	0.6	-2.1	3.3	0.66
ASA (3-4)	2.2	-0.5	4.9	0.11
Sex (F)	-1.5	-3.1	0.1	0.065
Stoma (Yes)	2.6	-0.8	6.0	0.31
Midline incision	-1.7	-3.0	-0.4	<0.011

## Discussion

To our knowledge, this is an extensive study investigating the use of TAP blocks in elective laparoscopic colorectal surgery, specifically using a combination of dexmedetomidine, dexamethasone, and bupivacaine [1]. We compared postoperative pain control in patients who underwent elective minimally invasive colorectal surgery and who received either local wound infiltration or TAP blocks. Additional postoperative outcomes, such as ileus and LOS, were also studied. We showed that TAP blocks using two adjuncts that prolong anesthesia effects were associated with lower MME at 24 hours post surgery, despite having similar VAS scores compared to patients who received local anesthetic alone. Moreover, TAP blocks were associated with decreased postoperative ileus and shorter LOS. The concomitant use of PCA in 102 (52.3%) of the study population did not affect our results.

Before the introduction of ERAS protocols, opioid analgesia was often the first-line for the management of postoperative pain. However, opioids are associated with nausea, vomiting, delirium, and prolonged recovery time for bowel function, all of which can lead to a longer LOS [17]. Efforts to achieve opioid-free analgesia are now widely supported and encouraged through the use of perioperative locoregional anesthesia techniques, such as local wound infiltration or TAP blocks. Recently, TAP blocks have been included in the ERAS Society’s recommendations for elective colorectal surgery to reduce postoperative opioid consumption [18]. However, this recommendation is supported by only moderate quality of evidence as it was based on several small randomized controlled trials (RCTs) that reported contradictory results. In two RCTs comparing local wound infiltration to TAP blocks consisting of local anesthetic alone, no differences were found in postoperative opioid consumption, VAS, and LOS between the two groups [19,20]. Notably, diverging from these findings, other studies suggest that TAP blocks may lead to lower pain scores, faster bowel recovery, and reduced total opioid intake postoperatively [21,22]. TAP blocks used in previous RCTs and meta-analyses predominantly featured local anesthetics alone. In contrast, this study utilized TAP blocks that included a combination of dexmedetomidine and dexamethasone as dual adjuncts to the local anesthetic (bupivacaine). As a result, direct comparisons between our results and others may be challenging.

Several systematic reviews have reported that dexamethasone and/or dexmedetomidine can serve as adjuncts to local anesthetics in TAP blocks and have been shown to prolong the anesthetic effect [8,9,23]. Dexamethasone is hypothesized to inhibit potassium channels, thereby reducing stimulus transmission in C-fibers [24]. In contrast, dexmedetomidine is believed to produce an analgesic effect by stimulating the local release of an enkephalin-like substance peripherally [25] and having a direct central effect on the locus coeruleus secondary to its systemic absorption [26]. Combining both dexamethasone and dexmedetomidine may have a synergistic effect on improving pain control, as each drug has its own mechanism of action.

The combination of these two agents during locoregional anesthesia has been well-studied in orthopedic surgery. Berger et al. demonstrated that adding both to a local anesthetic for an interscalene nerve block before shoulder surgery resulted in significantly prolonged analgesia and reduced postoperative opioid consumption compared to using local anesthesia alone [11]. A randomized trial conducted by Aliste et al. reported that combining both dexamethasone and dexmedetomidine with local anesthetic for ultrasound-guided infraclavicular blocks during upper limb surgery provided a longer duration of motor block and extended postoperative analgesia time compared to patients who received a block with only dexamethasone and local anesthetic [10]. Similar results have also been shown for lower limb blocks [27].

To date, only one study has investigated the use of both dexamethasone and dexmedetomidine as dual adjuncts to local analgesia in TAP blocks following minimally invasive colorectal surgery. This study compared bupivacaine TAP blocks with dexamethasone and dexmedetomidine to those with dexamethasone alone, showing that the dual combination resulted in significantly less opioid use up to 48 hours after surgery [12]. However, their sample size was very small, with only 55 patients randomized between the two groups. While our study population was nearly four times larger, with 105 patients receiving dexamethasone and dexmedetomidine in their TAP blocks, we were able to replicate similar results, demonstrating that TAP blocks with dexamethasone and dexmedetomidine caused significantly lower MME at 0 hours and 24 hours postoperatively. In addition, we showed that TAP blocks with dexamethasone and dexmedetomidine were associated with lower LOS and postoperative ileus. Admittedly, it is difficult to compare these two studies, as the methodologies used were quite different, and our study could only compare TAP blocks to local wound infiltration.

That said, since only a TAP block containing bupivacaine hydrochloride, dexamethasone, and dexmedetomidine was compared to local infiltration with bupivacaine hydrochloride, determining the benefit of dexmedetomidine is challenging. Ideally, this study should have compared the TAP block we used with another TAP block containing only bupivacaine hydrochloride and dexamethasone. However, due to the recent adoption of a TAP block at our institution that includes bupivacaine hydrochloride, dexamethasone, and dexmedetomidine, the number of patients who received a TAP block with only bupivacaine hydrochloride and dexamethasone was minimal. Therefore, this comparison was not feasible. This would have enabled us to observe the effects of dexmedetomidine when added to dexamethasone. This study has several limitations that should be acknowledged. First, its retrospective design introduces inherent risks of selection bias, unmeasured confounding, and limited control over data completeness and accuracy. Second, the absence of randomization and reliance on institutional practice patterns limit causal inference and preclude isolation of the independent effect of dexmedetomidine. Thirdly, relying on the VAS to assess pain levels is subjective and varies among patients, resulting in similar VAS scores despite lower MME [28]. However, similar to other previously published studies, postoperative opioid use (measured by MME) served as an objective indicator of pain levels after surgery. Finally, reliance on postoperative PCA, the use of which is determined at the anesthesiologist’s discretion at our institution, may have resulted in higher opioid consumption in both groups. Nonetheless, the difference in postoperative PCA use between the two groups was minimal and not statistically significant. Furthermore, the subgroup analysis excluding PCA patients indicated that TAP blocks were associated with lower MME 24 hours after surgery, shorter LOS, and pain scores comparable to those in the local wound infiltration group. The most significant limitation of this study was the comparative arm, which utilized only local wound infiltration since this was the standard method of postoperative analgesia at our institution before the adoption of TAP blocks. Lastly, the study is limited by its small sample size, which may affect the generalizability of the findings. Nevertheless, our study can be regarded as a pilot study that offers a foundation for developing a prospective trial comparing TAP blocks with dexamethasone alone added to local anesthetic versus the combination of both dexamethasone and dexmedetomidine added to local anesthetic.

## Conclusions

TAP blocks using bupivacaine, dexamethasone, and dexmedetomidine following laparoscopic colorectal surgeries are associated with decreased opioid consumption 24 hours after surgery, shorter LOS, and decreased postoperative ileus. These findings suggest that the addition of these dual adjuncts to local anesthetics in TAP blocks may enhance recovery and support the goals of ERAS protocols by promoting earlier mobilization and faster return of bowel function. While pain scores were similar between groups, the lower opioid requirements highlight the potential of this approach to minimize opioid-related adverse effects. Given the retrospective design and limited sample size of this study, these results should be interpreted cautiously. Nonetheless, our data provide preliminary evidence supporting the incorporation of TAP blocks with combined dexamethasone and dexmedetomidine into multimodal analgesia strategies. Future prospective, randomized controlled trials are warranted to confirm these benefits and to determine the optimal dosing and combination of adjuncts for maximal postoperative analgesia and recovery in patients undergoing laparoscopic colorectal surgery.
